# UBA1: At the Crossroads of Ubiquitin Homeostasis and Neurodegeneration

**DOI:** 10.1016/j.molmed.2015.08.003

**Published:** 2015-10

**Authors:** Ewout J.N. Groen, Thomas H. Gillingwater

**Affiliations:** 1 Euan MacDonald Centre for Motor Neurone Disease Research, University of Edinburgh, Edinburgh, UK; 2 Centre for Integrative Physiology, University of Edinburgh, Edinburgh, UK

## Abstract

Neurodegenerative diseases are a leading cause of disability and early death. A common feature of these conditions is disruption of protein homeostasis. Ubiquitin-like modifier activating enzyme 1 (UBA1), the E1 ubiquitin-activating enzyme, sits at the apex of the ubiquitin cascade and represents an important regulator of cellular protein homeostasis. Critical contributions of UBA1-dependent pathways to the regulation of homeostasis and degeneration in the nervous system are emerging, including specific disruption of UBA1 in spinal muscular atrophy (SMA) and Huntington's disease (HD). In this review we discuss recent findings that put UBA1 at the centre of cellular homeostasis and neurodegeneration, highlighting the potential for UBA1 to act as a promising therapeutic target for a range of neurodegenerative diseases.

## Trends

Disruption of protein homeostasis is an important feature of many neurodegenerative diseases. The E1 ubiquitin-activating enzyme UBA1 sits at the apex of ubiquitin pathways, playing a critical role in regulating protein homeostasis. UBA1 regulates a diverse range of cellular processes in the nervous system.

UBA1 contributes to the pathogenesis of several neurodegenerative diseases, including SMA and HD. In SMA, decreased UBA1 expression leads to perturbations in ubiquitin homeostasis, aberrant accumulation of downstream target proteins, and neuromuscular degeneration. In HD, UBA1 expression decreases over time, leading to selective accumulation of toxic forms of huntingtin protein in the brain.

UBA1 represents a novel and promising therapeutic target for the treatment of neurodegenerative diseases.

## Disruption of Protein Homeostasis in Neurodegenerative Disease

Neurodegenerative diseases are a common cause of disability and early death throughout global populations [1,2]. Although our understanding of the underlying pathogenic mechanisms has improved greatly over recent years, most neurodegenerative diseases currently remain untreatable and incurable. Considerable research efforts are therefore seeking new therapeutic targets capable of delaying or halting progression of these conditions. Although some neurodegenerative diseases have a simple monogenetic origin, a combination of sporadic and familial forms is far more common, generating considerable challenges for therapy development [3].

In contrast to the complex interplay of genetic and environmental factors underlying most neurodegenerative diseases affecting the human population [4], many of these conditions share a common molecular signature: disruption of protein homeostasis [5]. This often manifests as an accumulation of ubiquitylated proteins, with evidence for a robust contribution to disease pathogenesis in conditions such as **Parkinson's disease (PD)** (see Glossary), **Alzheimer's disease (AD)**, **Huntington's disease (HD)**, and **amyotrophic lateral sclerosis (ALS)** [6–9]. Importantly, disruption of protein homeostasis can also occur without aggregation of proteins, as illustrated in the case of **spinal muscular atrophy (SMA)** [10,11].

Correct protein degradation is required to maintain cellular homeostasis in all cells and tissues, including the nervous system, and is regulated by two main pathways: the ubiquitin–proteasome system (UPS) and autophagy. The UPS identifies and marks proteins for degradation by covalent binding of ubiquitin to one or more lysine residues of a target protein [12]. This reaction is mediated by an E1–E2–E3 enzymatic cascade that is similar for ubiquitin and other ubiquitin-like proteins (UBLs) such as NEDD8, SUMO, and IGS15 (Box 1) [13]. In addition, ubiquitylation of target proteins can regulate protein localization and function, independent of degradation [12]. Autophagy is a broad term used to describe the degradation of cytoplasmic components including proteins and organelles by lysosomes [14]. Cytoplasmic components are targeted for degradation by autophagic pathways by an E1–E2–E3 enzymatic cascade that is similar to that for ubiquitin [15]. Mutations in members of both pathways have been associated with several neurodegenerative diseases. These include mutations in the E3 ligase *PARKIN* and the deubiquitinating enzyme *UCHL1*, which are both associated with PD [16,17], and mutations in the autophagy receptor *SQSTM1* (p62) and *UBQLN2* (which has been implicated in both the UPS and autophagy), which are associated with ALS [18,19]. Similarly, loss of autophagy machinery or proteasome subunits in the nervous system leads to complex neurodegenerative phenotypes [20–22].

Whereas ∼40 E2-conjugating enzymes and hundreds of E3 ubiquitin ligases exist, mammalian cells express only two E1 ubiquitin-activating enzymes [13,23]. Because many degenerative diseases have been associated with distinct disease proteins, research targeting the UPS in these diseases has mainly focused on the more substrate-specific E2 and E3 enzymes [24]. Recent work, however, has specifically implicated the main upstream E1 ubiquitin-activating enzyme (UBA1) in a range of neurodegenerative diseases [10,11,25], suggesting a central role for UBA1 in the regulation of neurodegeneration. In this review we provide an overview of the canonical role of UBA1 in ubiquitin homeostasis and discuss how altered UBA1 function disrupts a range of core cellular and neuronal processes, including those affected in neurodegeneration. These observations lead us to propose a model whereby UBA1 represents a novel and promising therapeutic target for neurodegenerative disorders.

## UBA1 as a Key Regulator of Protein Homeostasis

The range of neurodegenerative diseases characterized by changes in protein homeostasis illustrates just how vital these pathways are for maintaining a healthy nervous system. Maintaining protein homeostasis requires correct tagging of target proteins with ubiquitin. The first step in achieving this involves the activation of ubiquitin by UBA1 (Box 1). UBA1 is a highly conserved protein that is expressed in two main isoforms of 1058 (UBA1a) and 1018 (UBA1b) amino acids. Expression of UBA1 is essential, as deletion of the *UBA1* gene has been shown to be lethal [26,27]. Although the FAT10-activating E1 enzyme UBA6 has also been shown to be capable of activating ubiquitin [28–30], the very high expression of UBA1 suggests that ubiquitin pathways do not depend on activation by UBA6 [29]. This puts UBA1 firmly at the center of the regulation of protein homeostasis.

In addition to activating and transferring ubiquitin, UBA1 can also activate and transfer the UBL NEDD8 under stress conditions (specifically, when cellular ubiquitin levels are low), which can lead to a mixture of NEDD8 and ubiquitin being conjugated to protein substrates [31]. In addition, it has been shown that UBA1 can be required for Atg8-dependent autophagy, although not through direct activation of the autophagy-associated UBL Atg8 [32]. In this case, UBA1 activity bypasses the E1 and E2 enzymes Atg7 and Atg3 that are normally required for autophagy, which suggests that, at least in certain tissues, crosstalk between UBA1 and autophagy pathways is possible. As described above, the E1 enzyme UBA6 is also capable of activating ubiquitin [29]. However, in contrast to UBA1, UBA6 is able to activate both ubiquitin and the UBL FAT10, but has so far been shown to be associated with only one specific E2 enzyme, USE1. This suggests that UBA6-mediated ubiquitin activation might be required for ubiquitylation of (a subset of) specific proteins rather than playing a central role in protein homeostasis [29]. For example, brain-specific depletion of UBA6 expression in mice leads to a range of neurodevelopmental and behavioral defects associated specifically with elevated levels of the E3 ligase Ube3a and decreased expression of known downstream targets [33]. Also, it has been shown that proteasomal degradation of UBA1 itself is regulated by UBA6 and USE1-mediated activation and conjugation of FAT10 to UBA1 [34]. These studies demonstrate that considerable crosstalk exists between different E1 pathways and reveal that the regulation of these processes and enzymes is more complex than previously thought.

The combination of activation and conjugation of ubiquitin and UBLs by multiple E1, E2, and E3 enzymes provides cells with practically limitless possibilities for fine-tuning the targeting of protein substrates toward specific cellular pathways and fates. Importantly, it also suggests an even broader role for UBA1 in ubiquitin and UBL activation and implicates UBA1 in the regulation of a wide range of cellular processes.

Early work investigating the cellular functions of UBA1 established it as an important regulator of cell cycle progression. Ubiquitylation and deubiquitylation of key cell cycle proteins, including histone H2A and p53, by numerous enzymes, including UBA1, is essential for cells to progress through the phases of the cell cycle [35–37] and therefore complete loss of UBA1 function has detrimental effects on cell cycle progression. Temperature-sensitive mutations revealed how loss of UBA1 function leads to an overall reduction in the levels of ubiquitylated proteins and protein degradation, causing cell cycle arrest [38,39]. Pharmacological inhibition of UBA1 prevented UPS-mediated degradation of the tumor suppressor protein p53, inhibiting progression through the cell cycle [35,36]. This requirement for UBA1 is further illustrated by its subcellular localization, which is intimately related to cell cycle progression. During G1 and G2 phase, UBA1 is almost exclusively nuclear, whereas in other mitotic phases it is present in both the nucleus and the cytoplasm [40]. How changes in the subcellular localization of UBA1 are functionally related to cell cycle progression remains to be further investigated.

**Figure 1:**
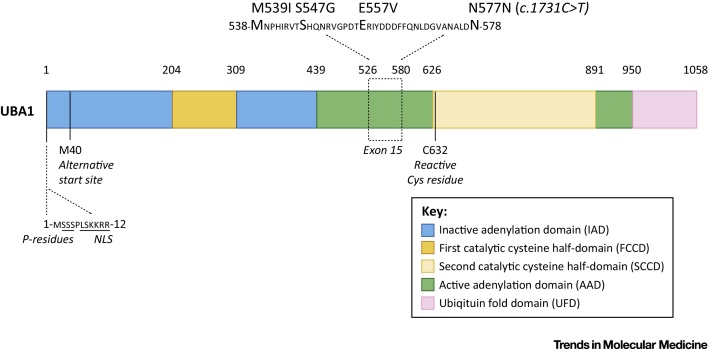
Domains of Ubiquitin-Like Modifier Activating Enzyme 1 (UBA1) and Genetic Variants Identified in X-Linked Spinal Muscular Atrophy (XL-SMA). The N-terminal half of UBA1 comprises an inactive adenylation domain (IAD) that surrounds the first catalytic cysteine half-domain (FCCD). The C-terminal half of UBA1 comprises an active adenylation domain (AAD) that surrounds the second catalytic cysteine half-domain (SCCD). The SCCD contains the reactive Cys residue that binds ubiquitin. The C-terminal ubiquitin fold domain (UFD) allows UBA1 to bind to E2 enzymes. When UBA1 is folded into its 3D structure, the FCCH and SCCH and the IAD and AAD are directly adjacent to each other [78,79] (Box 1). The Met residue at position 40 provides an alternative translational start site that leads to the expression of the UBA1b isoform of the protein. The UBA1a isoform-specific N-terminal sequence contains a nuclear localization signal (NLS) and Ser residues that can be phosphorylated (P residues). Mutations in UBA1 that have been shown to cause XL-SMA cluster in exon 15 of the protein. The domain structure in this figure is based on yeast [78] and mouse [79] UBA1 structural analysis. The specific amino acid positions in the figure are based on the mouse UBA1 sequence, as the mouse and human UBA1 protein sequences are more than 95% identical. The amino acids that are mutated in XL-SMA as well as the amino acid sequences that surround the borders of the various domains are all perfectly conserved between mouse and human UBA1 sequences.

UBA1 can be phosphorylated at several serine residues [41], again closely linked to cell cycle status; UBA1 phosphorylation is maximal during G2 phase [42]. Phosphorylation and nuclear localization are also related to the expression of the isoforms of UBA1, UBA1a and UBA1b. These isoforms differ in their translational start site, leading to an additional N-terminal 40 amino acids in UBA1a [42]. The first 11 amino acids of UBA1a (absent in UBA1b) contain a nuclear localization signal (NLS) and four serine residues essential for phosphorylation and nuclear localization (Figure 1). Non-phosphorylated UBA1a can still localize to the nucleus, but the NLS is required for efficient phosphorylation [42]. Interestingly, reduced phosphorylation of UBA1a in macrophages was shown to attenuate nucleotide excision repair deficiencies in terminally differentiated macrophages [43]. In addition, UBA1 was shown to be essential for protein ubiquitylation-mediated repair of double-strand DNA breaks [44]. These findings illustrate that UBA1 is also implicated in DNA repair pathways.

## UBA1 is Essential for Maintaining Neuronal Homeostasis

In neurons, local regulation of protein translation and degradation (including at distal cellular sites such as the synapse) is required for normal cellular function. The important contribution of the UPS to these pathways has been demonstrated for many different aspects of pre- and postsynaptic form and function, including growth cone guidance and synaptic transmission (see, for example, [45,46]). As UBA1 represents the initiating apex of UPS pathways in neurons, UBA1 is at the center of a range of cellular and molecular pathways required for the development and maintenance of a healthy nervous system. It is perhaps unsurprising, therefore, that disruption of UBA1 in neurons leads to compromised neuronal form and function. For example, inhibition of UBA1 leads to increased miniature and spontaneous synaptic currents at both excitatory and inhibitory synapses in cultured hippocampal neurons [47]. Likewise, UBA1 is required for axon development in *Drosophila*, where loss of UBA1 leads to pruning defects [48]. Interestingly, increased levels of UBA1 have also been associated with the **slow Wallerian degeneration (Wld**^**s**^**) phenotype** [49,50].

The studies discussed above reveal an important role for UBA1 in the regulation of numerous core cellular and molecular pathways highly relevant to neurons, including cell cycle status, developmental axon pruning, and neurotransmitter release. The range of cellular pathways implicated in UBA1 dysfunction therefore raises the strong likelihood that disruption of UBA1 levels and/or function contributes to pathological changes in the nervous system occurring during neurodegeneration. Importantly, several lines of evidence suggest that the timing and severity of UBA1 perturbations are likely to dictate the resulting phenotype. For example, studies in *Drosophila* have shown that partial loss of UBA1 leads to defects in apoptosis [51,52] whereas complete loss of UBA1 leads to cell cycle arrest [51] and tissue overgrowth in a non-cell autonomous manner [51,52]. Moreover, in *Caenorhabditis elegans* loss of UBA1 function at different developmental stages leads to a range of phenotypes including embryonic or larval lethality, decreased fertility in adult stages, and late-onset paralysis [26].

## UBA1 and the Regulation of Neurodegeneration

The importance of UBA1 for the maintenance of neuronal health and function has triggered considerable research efforts aiming to specifically investigate the extent to which this critical ubiquitylation enzyme contributes to neurodegenerative disease. Initial work with *Drosophila* models has shown that mutations associated with modest impairment of UBA1 function and expression [52] lead to a phenotype reminiscent of neurodegenerative disease. Flies that are homozygous for a UBA1 allele that represents a partial loss of function are characterized by a marked decrease in lifespan and severe locomotion deficits [53].

Specific links between UBA1 and neurodegenerative diseases affecting the human population have recently been established, with strong clinical and experimental data highlighting roles for UBA1 in SMA. Mutations in *UBA1* cause a rare form of SMA known as X-linked SMA (XL-SMA), a disease that is clinically similar to SMA but not caused by homozygous deletion of the *SMN1* gene [10,54,55]. Clinically, XL-SMA is characterized by muscle weakness associated with anterior horn motor neuron loss, **hypotonia**, and **areflexia**. Moreover, in contrast to SMA, XL-SMA is typically associated with **congenital contractures** and fractures [10,54,55]. Pathologically, widespread sensory and cerebellar abnormalities have been described, although the thalamic pathology that is often observed in *SMN1*-dependent SMA was absent [54]. Interestingly, all mutations that have so far been identified in XL-SMA cluster in exon 15 of the UBA1 gene (Figure 1). How these mutations lead to neurodegeneration is currently unknown, but it is likely that they are associated with impairment of UBA1 function, possibly due to altered methylation patterns of exon 15 [10].

Although XL-SMA represents a very rare form of SMA, recent work has demonstrated that UBA1 may also play an important role in the pathogenesis of more common, *SMN1*-dependent forms of the disease. Suppression of full-length survival motor neuron protein (SMN) was found to lead to widespread disruption of protein homeostasis in SMA mouse models, including a robust drop in levels of UBA1 protein [11]. Investigation of the molecular mechanisms linking SMN depletion with the regulation of UBA1 protein levels suggested a complex interaction involving multiple potential routes, including modifications in the splicing of *UBA1* and direct protein–protein interactions between UBA1 and SMN [11]. Experiments utilizing zebrafish revealed that replicating this suppression of UBA1 *in vivo* was sufficient to phenocopy the motor neuron axon defects observed in SMA-model zebrafish [11]. This supports the finding from earlier studies utilizing *Drosophila*, described above, which showed that targeting UBA1 was sufficient to generate a pronounced motor behavior phenotype and decrease survival [53]. Given that there is a growing body of evidence suggesting that the most severe forms of SMA involve pathological changes in a range of cells and tissues within and beyond the neuromuscular system [56], it was perhaps unsurprising that UBA1 defects were also identified across a broad range of body organs in SMA mice [11]. The contribution of UBA1 to SMA pathogenesis has even been demonstrated at the level of single cell types, where myelination defects in peripheral nerves [57] were found to occur due to reduced levels of UBA1 in Schwann cells [58].

Although UBA1 is strongly linked to the pathogenesis of SMA, recent findings suggest that its influence on neurodegeneration pathways extends beyond this condition. For example, it has been demonstrated that UBA1 acts as an important modifier of polyglutamine (polyQ) protein toxicity in a mouse model of HD [25]. Inhibition of UBA1 led to an increase in levels of mutant protein aggregates and with increasing age expression of UBA1 was found to decline. These findings suggest that decreased ability of UBA1 to degrade mutant protein correlates with increased accumulation of mutant protein species in affected tissues over time [25], identifying a potential role for UBA1 in neurodegenerative diseases that are characterized by their late onset. In addition, UBA1 knockdown has been shown to increase HD-associated polyQ protein aggregation in an siRNA screen in *C. elegans* [59]. Thus, UBA1 appears to have the capacity to influence neurodegeneration in conditions manifesting primarily in the early stages of life (SMA) as well as those that are associated with advancing age (HD).

Alongside these direct links between UBA1 and SMA/HD, indirect evidence from protein-interaction and -modifier studies provides further experimental support indicating a potentially important role for UBA1 in regulating neurodegeneration pathways relevant to a broad range of diseases. For example, UBA1 modifies the toxicity of a specific *Tau* genetic mutant in *Drosophila* [60]. When interacting proteins of the ALS-associated protein FUS were studied, UBA1 was identified as preferentially binding to and interacting with an ALS-causing FUS mutant but not wild-type FUS [61]. Moreover, exposure of mouse and rat models to pesticides implicated in idiopathic PD has been shown to lead to increased expression of the PD disease protein alpha-synuclein as well as selective damage to dopaminergic neurons and locomotion defects [62,63] that are specifically associated with UBA1 but not proteasome inhibition [64]. Finally, in the cytosolic fraction of AD brain samples, the expression and activity of UBA1 was strongly reduced [65].

**Figure 2:**
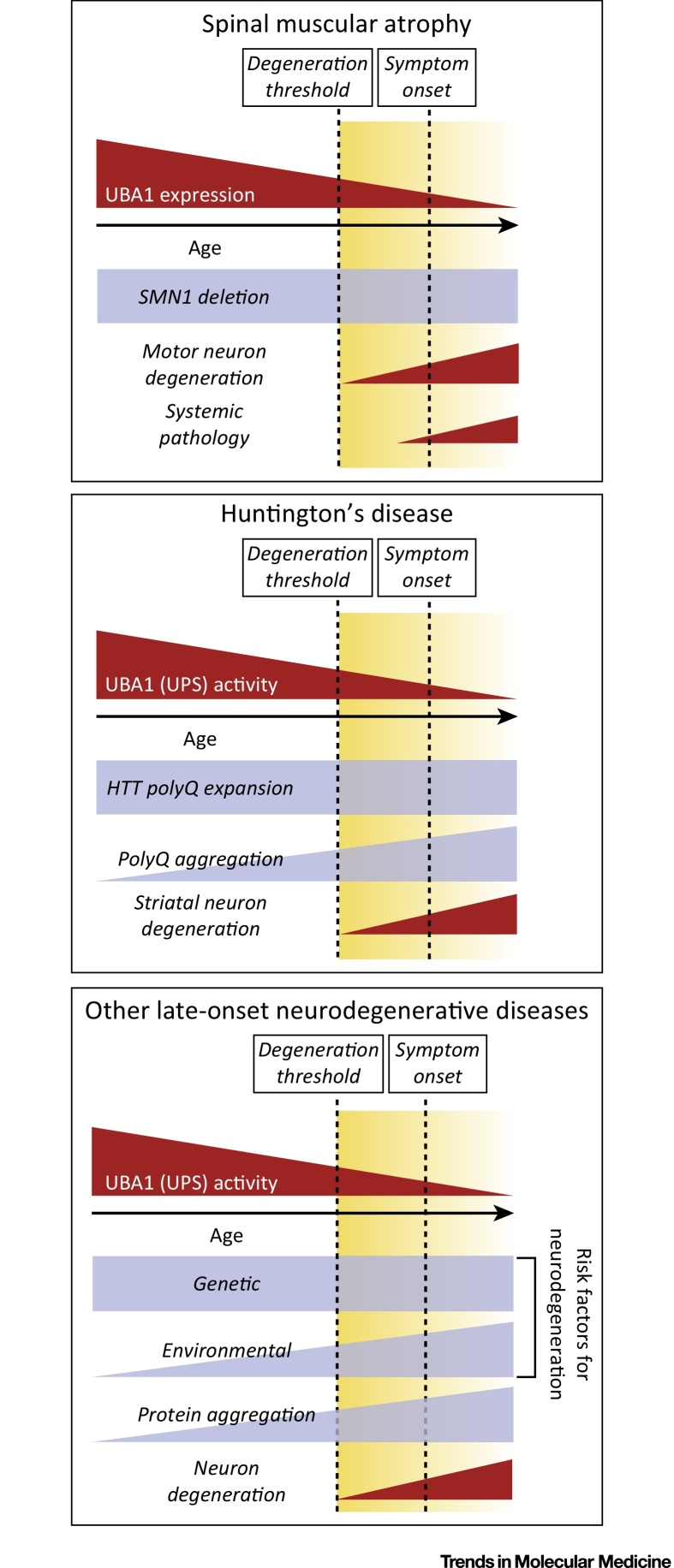
Key Figure: Contribution of Ubiquitin-Like Modifier Activating Enzyme 1 (UBA1) to Neurodegenerative Disease

Taken together, the studies discussed above provide experimental evidence linking altered levels or activity of UBA1 with pathogenic events underlying a range of neurodegenerative diseases, including HD, PD, ALS, and SMA. This places UBA1 at the center of a molecular ‘hub’ capable of modulating neurodegenerative pathways in the nervous system triggered by a diverse range of genetic defects and/or environmental factors (Figure 2, Key Figure). However, this model raises something of a quandary: how can changes in the levels and/or activity of a ubiquitously expressed core E1 enzyme contribute to neurodegenerative conditions where, more often than not, specific cell types are susceptible (e.g., lower motor neurons in SMA, striatal neurons in HD)? Mutations in the *UBA1* gene in humans disrupt UBA1 levels and function throughout all cells and tissues of the body but manifest as an early-onset neurodegenerative disease where lower motor neurons are particularly affected (XL-SMA) [10,54,55]. This is consistent with findings in *Drosophila*, where motor neurons appear to be particularly sensitive to perturbations in UBA1 [53]. This suggests that motor neurons (particularly large neurons with a requirement to support very long axonal processes and distal synaptic terminals) are particularly susceptible to perturbations in ubiquitin homeostasis. This model is supported by the finding that low levels of SMN protein in SMA lead to suppression of UBA1 protein levels throughout the body, but again lower motor neurons are the primary pathological target [11], in agreement with the ‘threshold model’ hypothesis for SMA [66]. For neurodegenerative conditions caused by genetic or environmental factors not associated with global perturbations in UBA1 levels or function, it is perhaps most likely that targeting of restricted neuronal populations results from differential susceptibility of neuronal populations to the initial triggering ‘insult’ itself, with UBA1-dependent pathways subsequently engaged downstream. For conditions where severe disruption of protein homeostasis results from the formation of protein aggregates, this is likely to disrupt UBA1-mediated regulation of ubiquitin homeostasis, although the mechanism underlying this disruption is currently unclear. It is possible that UBA1 localization in neurons is disrupted, as has been shown in AD patient samples [65]. Moreover, UBA1 might be sequestered into disease-associated protein aggregates, as has been shown for **Lewy bodies** in models of PD [67]. Finally, as overall UPS function decreases with advancing age [68], UBA1 function might eventually fall below a critical threshold level required to maintain protein homeostasis, as was illustrated specifically for polyQ protein aggregation in HD [25].

## UBA1 as a Therapeutic Target for Neurodegenerative Disease

The fundamental role played by UBA1 in regulating cellular homeostasis and neurodegeneration suggests that targeting of UBA1 and/or its downstream pathways may represent a potential therapeutic approach to slow or halt the progression of several neurodegenerative conditions. Here we discuss current approaches that have been used to modulate UBA1 and UPS function and suggest possible future strategies to develop UBA1-based therapies.

The UPS has long been considered an attractive therapeutic target for conditions such as cancer, as many tumors are known to show aberrant protein ubiquitylation patterns and disrupted control of cell cycle progression [69]. For example, bortezomib and carfilzomib, both inhibitors of the 26S proteasome, are FDA-approved treatments for multiple myeloma. Their use illustrates how the targeting of even a nonspecific part of the UPS can be a safe and efficient way to treat disease, although not all patients respond to individual treatments and they may develop resistance over time [70]. UBA1-targeted therapies are also being developed in this regard, mainly focusing on small-molecule inhibitors of UBA1 (reviewed in [71]) targeting various aspects of UBA1-mediated ubiquitin activation [72]. It is important to note, however, that the studies detailed above show a strong link between reduced levels/activity of UBA1 and neurodegenerative phenotypes. Thus, one potential side effect of systemic suppression of UBA1 activity for cancer treatment could be an increased risk of, or susceptibility to, neurodegenerative disease.

In contrast to the development of UBA1 inhibitors for the treatment of cancer, upregulation of UBA1 levels and/or activity is likely to be required for the treatment of neurodegeneration. However, this approach has yet to be extensively explored and therapeutic tools remain limited. Importantly, from a safety perspective, several lines of evidence suggest that high levels of UBA1 are safe and well tolerated. For example, levels of UBA1 are increased by ∼40–60% in the nervous system of mice carrying the neuroprotective *Wld*^*s*^ mutation [49,50] and these mice show no overt phenotype (with normal behavior, lifespan, and organ histopathology and no evidence for increased oncogenic activity). Moreover, UBA1 comprises ∼2% of the total protein content of a cell [73,74] and at steady-state levels the UBA1-specific E2 enzymes CDC34 and CDC34B are fully charged with ubiquitin, which indicates that there is sufficient active E1 to maintain ubiquitin-charged E2 enzymes [29]. When sufficient levels of UBA1 are available, the interaction of E2-ubiquitin with E3 ligases therefore limits the rate of ubiquitylation [29]. This suggests that cells can tolerate high basal levels of UBA1 and that further upregulation of UBA1 levels may be physiologically tolerable.

Although therapeutically relevant strategies to increase levels of UBA1 are not currently available, several approaches may be worthy of consideration. For example, gene therapy-based approaches could be used to increase UBA1 expression levels in a temporally controlled, targeted manner. Alternatively, small-molecule screens could be employed to identify compounds capable of increasing UBA1 levels (by modulating transcription/translation or protein stability) or activity (for example, by providing favorable cellular conditions for UBA1 to activate ubiquitin). Moreover, targeted inhibition of UBA6-mediated degradation of UBA1 [34] is likely to be an efficient method to increase UBA1 levels.

As UBA1 depletion leads to a reduction in cellular ubiquitin pools [11], another therapeutic strategy could be to target the restoration of free ubiquitin levels by increasing the activity of deubiquitination enzymes (DUBs). When UBA1 is inhibited, the DUB UCHL1 is strongly upregulated [75] and inhibition of UCHL1 aggravates rather than ameliorates disease in a mouse model of SMA [75]. Moreover, subpopulations of motor neurons in a mouse model of ALS that are resistant to degeneration are characterized by high levels of UCHL1 expression [76]. This indicates that increasing activity of specific DUBs could also be an interesting therapeutic approach for diseases that are characterized by disruption to protein homeostasis.

Finally, an alternative approach for the development of therapies targeting UBA1-mediated neurodegeneration is to consider key substrate proteins that become differentially ubiquitylated downstream of perturbations in UBA1 [77]. One good example of where this approach has already shown considerable promise is for the treatment of SMA. Depletion of UBA1 in SMA resulted in decreased overall ubiquitylation, and ultimately accumulation, of beta-catenin protein, leading to increased beta-catenin signaling activity [11]. Pharmacological inhibition of beta-catenin robustly suppressed SMN-dependent and UBA1-dependent neuromuscular pathology *in vivo* [11], thereby highlighting the therapeutic potential of identifying and then targeting protein modifications occurring downstream of UBA1. Similar screens aimed at identifying UBA1-dependent target proteins in affected cell populations in other neurodegenerative conditions (e.g., HD, ALS, PD) may therefore also serve to generate novel therapeutic targets and development strategies.

## Concluding Remarks

Neurodegenerative diseases are common and severely disabling conditions that often lead to premature death. Disruption of protein homeostasis is a common feature of many such diseases and although further research is required (see Outstanding Questions) increasing evidence places UBA1 as a central regulator of these pathways. Therapeutic targeting of UBA1, or its downstream disease-specific targets, may therefore generate potential new avenues for slowing or halting disease progression in a broad spectrum of disorders.

Outstanding Questions How do disease-specific genetic and environmental triggers cause modifications to UBA1 expression and/or function?

Are there further disruptions in the expression of specific parts of the UPS downstream of UBA1 (E2, E3 enzymes)?

To what extent does disruption of UBA1 mediate neurodegeneration in diseases where only indirect links currently exist (such as AD, PD)?

What are efficient and safe ways of elevating UBA1 expression and/or activity *in vivo*, and does this provide a viable novel therapeutic approach for neurodegenerative diseases?

## Figures and Tables

**Figure I:**
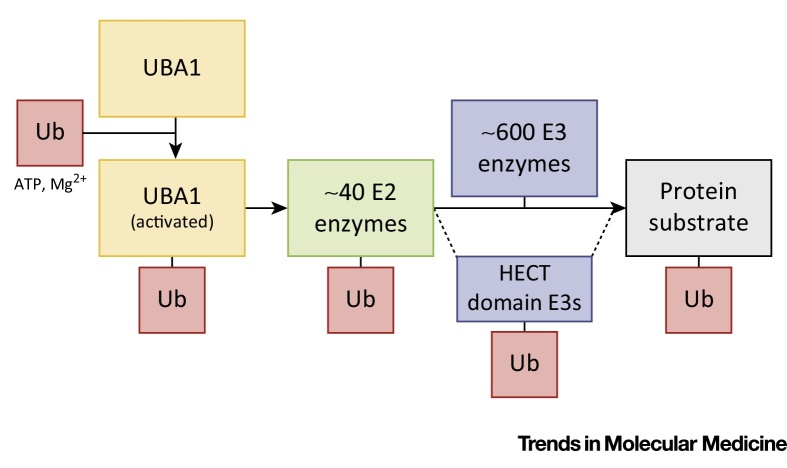
Simplified Representation of the E1–E2–E3 Ubiquitin Activation Pathway. In an ATP- and Mg^2+^-dependent reaction, ubiquitin-like modifier activating enzyme 1 (UBA1) activates ubiquitin and transfers it to one of ∼40 E2 conjugating enzymes. Subsequently, one of hundreds of E3 ligases facilitates the transfer of ubiquitin from the E2 enzyme to specifically mono- or polyubiquitylate a protein substrate. In addition, HECT E3 ligases are able to directly bind ubiquitin and thereby directly ubiquitylate protein substrates.

## Box 1 The E1–E2–E3 Ubiquitin Activation Pathway

Ubiquitin is transferred to its target proteins by an enzymatic cascade that involves three types of enzyme and is similar for ubiquitin and UBLs such as NEDD8, SUMO, and ISG15 as well as the two ubiquitin-like autophagy activation pathways (Figure I). The main initiator of the activation, conjugation, and ligation of ubiquitin to target protein substrates is the E1 ubiquitin-activating enzyme UBA1. In the first step of ubiquitin activation, one of the acetylation domains of UBA1 binds ubiquitin, ATP, and Mg^2+^ while the other acetylation domain is used to stabilize this complex [78]. The C terminus of ubiquitin is acyl-adenylated, allowing the reactive cysteine residue of UBA1 to attack this bond and produce a thioester link between UBA1 and ubiquitin. In a second ATP- and Mg^2+^-dependent reaction, UBA1 adenylates a second ubiquitin molecule, which leads to double loading of UBA1 with two ubiquitin molecules [80]. The double loading of UBA1 with two ubiquitin molecules enables the transfer of ubiquitin to E2-conjugating enzymes by providing a favorable conformation of the UBA1–ubiquitin complex [13]. The activated ubiquitin is subsequently transferred to one of ∼40 E2 enzymes, in a reaction where E1 and E2 enzymes physically interact to generate a similar thioester reaction. The E2–ubiquitin complex and the protein targeted for ubiquitylation are subsequently brought together by E3 ligases. E3 enzymes facilitate the transfer of activated ubiquitin from the E2 enzyme to the target protein. Moreover, a subtype of E3 ligases (HECT domain E3 ligases) bind ubiquitin through a reactive cysteine residue in their HECT domain and thus transfer ubiquitin to their protein substrate directly [81]. Hundreds of E3 enzymes exist and it is this step that provides specificity to the ubiquitin conjugation reaction [74]. The cycle of E1 activation and transfer of ubiquitin to E2 enzymes can be repeated leading to polyubiquitylation of substrate proteins, either through linking ubiquitin to one of the seven Lys residues or the initiating Met residue in ubiquitin itself (creating polyubiquitin chains) or by conjugating ubiquitin onto another Lys residue in the substrate protein. The site of protein ubiquitylation and the Lys residue at which polyubiquitin chains are formed are important determinants of the fate of the protein substrate being (poly)ubiquitylated [82].

## Glossary

Alzheimer's disease (AD): a neurodegenerative disease characterized by the extensive loss of neurons and synapses throughout the cortex and in certain subcortical regions and prominent neuropathology comprising beta-amyloid- (amyloid plaques) and tau- (neurofibrillary tangles) positive protein aggregates. The cause of AD is complex and is thought to involve genetic as well as environmental risk factors.
Amyotrophic lateral sclerosis (ALS): a neurodegenerative disease characterized by the degeneration of upper and lower motor neurons and the presence of polyubiquitylated, TDP43-positive protein aggregates. Mutations across a range of genes have been shown to cause ALS, although the vast majority of ALS patients have a sporadic form of the disease that is complex and thought to involve a combination of genetic and environmental risk factors.
Areflexia: the absence of normal reflexes.
(Congenital) contractures: the presence at birth of contracted or abnormally short muscles.
Huntington's disease (HD): a neurodegenerative disease characterized by the degeneration of striatal neurons and caused by a polyglutamine (polyQ) repeat expansion in the gene encoding huntingtin (*HTT*). Pathologically, HD is characterized by intraneuronal and intranuclear inclusion bodies that are positive for mutant HTT protein and, often, ubiquitin.
Hypotonia: low resistance to stretch or amount of tension in muscles, often related to reduced muscle strength.
Lewy body: eosinophilic, cytoplasmic protein aggregates found in several neurological diseases including PD and Lewy body dementia.
Parkinson's disease (PD): a neurodegenerative disease characterized by the degeneration of neurons in the substantia nigra and the presence of Lewy body and alpha-synuclein pathology. Several genetic forms of PD exist but most cases are idiopathic.
Slow Wallerian degeneration (Wld^s^) phenotype: a spontaneous mutation in mice leading to expression of a protein linking the N-terminal part of Ube4b to Nmnat1. This fusion protein provides substantial protection against Wallerian degeneration and this mouse model has therefore been used extensively to study neurodegeneration and neuroprotection *in vivo*.
Spinal muscular atrophy (SMA): a childhood-onset neurodegenerative disease characterized by the degeneration of lower motor neurons; >95% of SMA cases are caused by homozygous deletion of the s*urvival motor neuron 1* (*SMN1*) gene.
